# Finger Length Ratios in Serbian Transsexuals

**DOI:** 10.1155/2014/763563

**Published:** 2014-05-20

**Authors:** Svetlana Vujović, Srdjan Popović, Ljiljana Mrvošević Marojević, Miomira Ivović, Milina Tančić-Gajić, Miloš Stojanović, Ljiljana V. Marina, Marija Barać, Branko Barać, Milena Kovačević, Dragana Duišin, Jasmina Barišić, Miroslav L. Djordjević, Dragan Micić

**Affiliations:** ^1^Clinic for Endocrinology, Diabetes and Metabolic Diseases, Clinical Centre of Serbia, Faculty of Medicine, University of Belgrade, Dr Subotića 13, 11000 Belgrade, Serbia; ^2^Faculty of Medicine, University of Banja Luka, Bosnia and Herzegovina; ^3^Clinic for Psychiatry, Clinical Centre of Serbia, Serbia; ^4^University Childrens Hospital, Tiršova 10, 11000 Belgrade, Serbia

## Abstract

Atypical prenatal hormone exposure could be a factor in the development of transsexualism. There is evidence that the 2nd and 4th digit ratio (2D : 4D) associates negatively with prenatal testosterone and positively with estrogens. The aim was to assess the difference in 2D : 4D between female to male transsexuals (FMT) and male to female transsexuals (MFT) and controls. We examined 42 MFT, 38 FMT, and 45 control males and 48 control females. Precise measurements were made by X-rays at the ventral surface of both hands from the basal crease of the digit to the tip using vernier calliper. Control male and female patients had larger 2D : 4D of the right hand when compared to the left hand. Control male's left hand ratio was lower than in control female's left hand. There was no difference in 2D : 4D between MFT and control males. MFT showed similar 2D : 4D of the right hand with control women indicating possible influencing factor in embryogenesis and consequently finger length changes. FMT showed the lowest 2D : 4D of the left hand when compared to the control males and females. Results of our study go in favour of the biological aetiology of transsexualism.

## 1. Introduction

Gender dysphoria is characterized by suffering from a strong, persistent discomfort between biological sex and experienced-expressed gender, with significant impairment in interpersonal, familial, social, professional, and other important areas of functioning [1]. Since 1964, when Harry Benjamin defined transsexualism, many etiological hypotheses were suggested. The cause of transsexualism remains unclear. The hypothesis that atypical prenatal hormone exposure could be a factor in the development of the transsexualism was examined by establishing whether an atypical pattern of digit length could be one of these manifestations [2]. Largest ever study of transsexual genetic, which compared the length of androgen receptor gene, the gene which is known to make circulating testosterone less effective, shown longer androgen receptor gene in male to female (MF) transsexuals. Less potent testosterone could affect the development of the brain “under masculinization” and make it more structurally similar to female brain (Prince Henry's Institute). This study is under criticism of many other scientific groups and require further examinations.

Sexual orientation in humans may be influenced by levels of prenatal sex steroids which canalize neuroendocrine development. As well, some lines of evidence for sexual-orientation-related differences in somatic markers of prenatal sex hormones support this view [3].

Recent attention has been paid to gender specific patterns of asymmetry in paired bilateral traits. Sexual dimorphism on digit length ratio is a feature common to many mammals [4]. Previous studies indicated that the fingers in the adult human hand differ in length and in distal extent in the clear majority of males [5]. The distal extent of the ring finger (4D) tends to be relatively greater, using the middle finger as standard, than the index finger (2D) in men. So, men have smaller 2D : 4D ratio compared to women. It was hypothesized that finger length pattern development might be affected by early androgen exposure. There is evidence that the ratio of the length of 2nd and 4th digits (2D : 4D) associates negatively with prenatal testosterone and positively with prenatal estrogens [6].

During early fetal life the digits of the hands are similar in length. Subsequently, under hormone exposure (predominantly androgens), differentiation leads to a pattern of unequal finger lengths, described by Peters et al. as the finger-digit length pattern [7]. The sexual dimorphism is determined as early as the 14th week of fetal life [8]. A significant negative association between 2D : 4D ratio and fetal testosterone/fetal estradiol ratio was found in amniotic fluid. These findings lent support to an association between low 2D : 4D and high levels of free testosterone relative to free estradiol and high 2D : 4D with low free testosterone relative to free estradiol [9]. Prenatal androgen appears to be important in the development of 2D : 4D sex difference, since it has been reported in children as young as 2 years old and since human exposed to supernormal androgen level display a smaller 2D : 4D ratio. The common control by the Hox genes of the differentiation of both the urogenital system and the appendicular skeleton has been proposed as an explanation for the recent finding that fluctuating asymmetry and the 2D : 4D are both associated [10, 11].

The androgen receptor gene contains a domain, which includes a variable number of CAG sequences and alleles with low number of CAG repeats show high transactivation activity when complexed with testosterone. Low number of CAG repeats and low 2D : 4D are both associated with high sperm number and protection against breast cancer. This suggests that CAG number and 2D : 4D are correlated; that is, low CAG number and low 2D : 4D indicate high activation of androgen-responsive genes [2].

Second and forth digit ratio was also found to be correlated with sexual orientation, left hand preference, fetal growth, Asperger syndrome, sperm count, autism, breast cancer in women, and myocardial infarction in men [9, 12].

Such an indirect parameter, as digit-length, can partly reflect hormone milieu during fetal life.

In the last 20 years Belgrade gender team followed up 250 transsexuals. While many previous studies founded higher incidence of male to female transsexuals, compared to female to male transsexuals, our study shown the equal number [13]. So, it was interesting to compare the digit length ratio (2D : 4D) in our country and compare them with the results from other studies and controls.

The aim of this study was to determine whether adult sexually dimorphic physical traits (like finger length ratio) relate to traits that are largely determined in utero, namely, whether reduced androgenization in utero during fetal development influenced occurrence of female to male transsexuals (FMT) and reduced androgens in male to female transsexuals (MFT). In addition, we wanted to assess difference in 2D : 4D ratio between transsexuals and controls.

## 2. Subjects and Methods

The tested groups were divided into the following:

- male to female transsexuals (MF): 42 subjects.
- female to male transsexuals (FM): 38 subjects.

Main characteristics of transsexuals are shown in Table 1.

The diagnosis of gender identity disorder was made by consensus of two board certified psychiatrists, according to the criteria of the 4th edition of the Diagnostic and Standard Manual of Mental Disorders [1].

They were, otherwise, healthy individuals:

(III) male controls (MC): 45 subjects, 30 ± 3.4 years of age, BMI 24 ± 3.2 kg/m^2^.
(IV) female controls (FC): 48 subjects, 28 ± 4.1 years of age, BMI 22.4 ± 3.1 kg/m^2^, with regular menstrual cycles.

Manning et al. [14] found that 2D : 4D from photocopies tended to be lower than that from direct measurements. Finger length differences could result from the shapes of fat pads at the tips of the fingers and these may be dependent on sex and sexual orientation. So, we made the decision to make a more precise measurement of digit lengths by X-rays at the ventral surface of the both hands from the basal crease of the digit to the tip using vernier caliper measuring to 0.05 mm, according to the standard published procedure and recommended guidelines of Bergsma and Feingold [15]. This measurement is known to show high degree of repeatability. Intraobserver variability in measurement technique was 0.01%. All measures were done in transsexuals prior to hormone reassignment therapy.

At the time of the research the medical authorities in Serbia did not require approval of the Ethic Committee. Participation in the study was voluntary and anonymous.

We have used parametric test (unpaired *t*-test, simple linear and multiple regression test) for all analysis. Means and standard errors were reported as measures of central tendency and dispersion. Statistical analysis was performed with ANOVA, Kruskal-Wallis and Wilcoxon test.

## 3. Results

**Figure 1:**
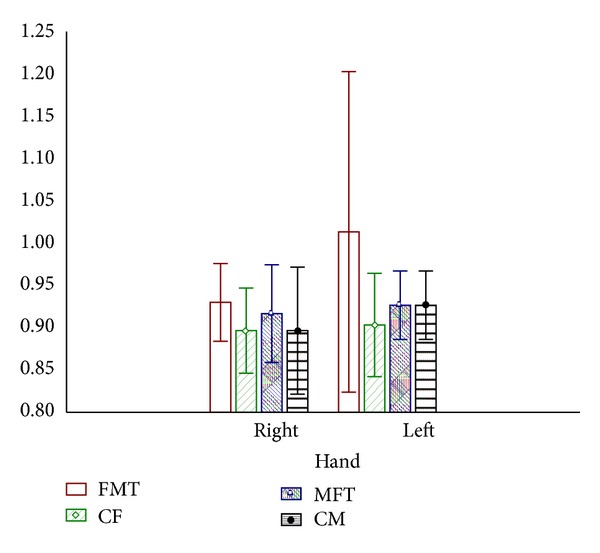
Finger length patterns in transsexuals and controls. FMT: female to male transsexuals. CF: control females. MFT: male to female transsexuals. CM: control males.

Results of the 2D : 4D ratio in male to female transsexuals, female to male transsexuals, and control males and females are shown on Figure 1.

Our study found larger 2D : 4D for right hand in control males, compared to left hand (0.928 versus 0.935). Control female exhibited, as well, larger 2D : 4D for right hand, compared to left hand (0.921 versus 0.945). Control males left hand ratio 2D : 4D is lower (0.935) than in female left hand control (0.945) while there were no differences for the right hand (0.928 versus 0.921).

No differences were found between MFT and control males in 2D : 4D (0.935 : 0.935). MFT shown similar 2D : 4D of the right hand with control women (0.92 versus 0.921) indicating some possible etiological factor influencing period of embryogenesis and finger length changes. Interestingly, FMT shown the lowest 2D : 4D ratio of the left hand compared to the ratio in control males and females (0.926 versus 0.935 versus 0.945).

## 4. Discussion

The etiology of transsexualism is not yet clarified. Many hypothesis exist and this study shown some more data in examining some indirect parameters of early androgen exposure of the sexual dimorphic brain region and changes of finger length ratio.

From the period when Swaab discovered changes in a special brain nucleus differences between male and female transsexuals and controls attention was paid on all other body characteristics.

Kallai et al. [16] found that the 2D : 4D ratio was associated with and asymmetry in the hippocampal subregion. Smaller volume of the left side was found in posterior part of the hippocampus in female with a low (masculine type) 2D : 4D ratio. Thus development of the middle and posterior regions of the hippocampal formation may respond in opposite ways to prenatal levels of testosterone. Such a difference was not detected in some other brain regions. Our study confirmed that FMT had the lowest left hand 2D : 4D compared to control males and control females (0.926 versus 0.928 versus 0.945).

Study of Manning et al. [17] showed that 2D : 4D in right and left hands had a sexually dimorphic pattern. They found that in males 2D : 4D was 0.98 meaning that fourth digit tended to be longer than second while in females the ratio tended to be 1.0 meaning equal length. Our study indicated the ratio of 0.935 in control male left hand, while in control females left hand it was 0.945. Normative values of male mean 2D : 4D ratio vary between 0.94 and 1.0 across population [9]. We found 2D : 4D male ratio (right hand 0.928 versus left hand 0.935). According to Buck et al. study [18] the male ratio 2D : 4D was 0.918 and female 0.927. Larger sex differences were found for the right hand of males indicating that the right hand 2D : 4D is more sensitive to fetal androgens than the left hand ratio [19].

Schneider et al. [20] found 2D : 4D in MFT similar to that in control female which is consistent with our findings for the right hand (0.920 versus 0.91) confirming lower androgenization effects in MFT during embryogenesis.

FMT had the lowest left hand 2D : 4D compared to control females and control males (0.926 versus 0.945 versus 0.945) confirming the hypothesis of androgenization of female brain during prenatal period in FMT.

Peters et al. [5] have shown that the sexual dimorphism in finger measures is more strongly expressed in the distal extent of finger tips than in the length of finger. Smaller between-finger differences were found for females than for males. Lesser distal extent of the index finger, relative to the middle finger, was found in males than in females.

Nevertheless, Bang et al. [11] confirmed that finger length measurements do not have power to predict the testicular function in adult men.

Meta-analysis of accumulates evidence of effects of functional androgen receptor gene variants and 2D : 4D does not support initial evidence [21].

The present data suggest an early organizational effect of sex hormones through the association between body shape and finger length patterns. Also, these data draw attention to difficulties in the interpretation of results when somatic features are employed as biological markers.

## 5. Conclusion

Transsexualism in humans is biological in origin. Our findings support a biological etiology of MFT implicating decreased prenatal androgen exposure in MFT. 2D : 4D could be potentially used as a marker for prenatal androgen exposure.

## Figures and Tables

**Table 1:** Some characteristics of transsexuals.

Characteristics	Male to female	Female to male
Years of age	28.5 ± 3.6	34.3 ± 4.6
Weight (kg)	65.5 ± 10.8	69.1 ± 11.3
Hight (cm)	184.8 ± 12.3	168.8 ± 10.2
BMI (kg/m^2^)	24.2 ± 2.6	22.3 ± 3.7
Age at request (years)	23.5 ± 1.2	27.0 ± 3.4
Time since operation (years)	1.2 ± 2.3	1.4 ± 3.1
Mother's age (years)	26.0 ± 11.2	26.2 ± 20.3
Father's age (years)	30.7 ± 8.2	32.9 ± 10.1
